# Adjuvant endocrine therapy in pre- versus postmenopausal patients with steroid hormone receptor-positive breast cancer: results from a large population-based cohort of a cancer registry

**DOI:** 10.1007/s00432-015-2025-z

**Published:** 2015-08-08

**Authors:** E. C. Inwald, M. Koller, M. Klinkhammer-Schalke, F. Zeman, F. Hofstädter, P. Lindberg, M. Gerstenhauer, S. Schüler, O. Treeck, O. Ortmann

**Affiliations:** 1 Department of Gynecology and Obstetrics, University Medical Center Regensburg, Landshuter Straße 65, 93053 Regensburg, Germany; 2 Center for Clinical Studies, University Hospital Regensburg, Franz-Josef-Strauß-Allee 11, 93053 Regensburg, Germany; 3 Tumor Center Regensburg e.V., University of Regensburg, Josef-Engert-Straße 9, 93053 Regensburg, Germany; 4 Johannes Kepler University Linz, Altenberger Straße 69, 4040 Linz, Austria

**Keywords:** Endocrine therapy, Steroid hormone receptor, Breast cancer, Overall survival, Cancer registry

## Abstract

**Purpose:**

Adjuvant endocrine therapy (ET) is indicated in patients with steroid hormone receptor (HR)-positive breast cancer. The aim of this study was to evaluate the quality of HR determination and adjuvant endocrine treatment of breast cancer patients in a large cohort of more than 7000 women by analyzing data from a population-based regional cancer registry.

**Methods:**

Data from the Clinical Cancer Registry Regensburg (Bavaria, Germany) were analyzed. Female patients with primary, nonmetastatic invasive breast cancer who were diagnosed between 2000 and 2012 (*n* = 7421) were included. HR-status was available in 97.4 % (*n* = 7229) of the patients. This data set (*n* = 7229) was used for subsequent statistical analyses.

**Results:**

Since 2009, almost a complete rate of 99.6 % of analyzed HR-status was achieved. In sum, 85.8 % of the patients (*n* = 6199) were HR-positive, whereas 14.2 % (*n* = 1030) were HR-negative. Overall, 85.3 % (*n* = 5285) of HR-positive patients received ET either alone or in combination with chemotherapy (CHT) and/or trastuzumab. The majority of premenopausal patients received CHT plus ET (716 patients, 52.3 %). In postmenopausal patients, the most frequent systemic therapy was ET alone (2670 patients, 55.3 %). Best overall survival (OS) was found in HER2-/HR-positive patients receiving CHT plus ET plus trastuzumab (7-year OS rate of 97.2 % in premenopausal patients versus 86.9 % in postmenopausal patients). Premenopausal patients had a reduced benefit from additional CHT than postmenopausal patients. Premenopausal patients receiving only ET had a 7-year OS rate of 95.3 % compared to 92.7 % of patients receiving CHT plus ET. In contrast, postmenopausal patients treated with CHT plus ET had a 7-year OS rate of 84.0 % in comparison with those patients receiving only ET with a 7-year OS rate of 81.7 %.

**Conclusions:**

Analysis of HR in patients with early breast cancer achieved a very high quality in recent years. The vast majority of HR-positive patients received ET, and this guideline-adherent use improved OS. Inverse effects of the CHT plus ET combination in premenopausal versus postmenopausal patients and a still existing minority of patients not receiving guideline-adherent treatment should be further investigated in future studies.

## Introduction

In addition to surgery and irradiation as local therapies, almost all patients with early breast cancer receive adjuvant systemic medical treatments. These may include three components: chemotherapy (CHT), endocrine therapy (ET), and antibody therapy. Provided that breast cancer tissue expresses estrogen (ER) and/or progesterone (PR) receptors, adjuvant ET is indicated (Melcher et al. 2012). Adjuvant treatment with tamoxifen or aromatase inhibitors (AIs) leads to a reduction in both recurrence-free and overall survival (OS) (Early Breast Cancer Trialists’ Collaborative Group (EBCTCG) et al. 2005; Baum et al. 2002; Thürlimann et al. 2005). Current state of the art treatment for women with endocrine-responsive early breast cancer is an adjuvant ET for at least 5 years (Kreienberg et al. 2013; Untch et al. 2013; Lux et al. 2013). The type of ET depends on ovarian function. For premenopausal patients, the standard ET is 5 years of tamoxifen monotherapy (EBCTCG et al. 2005). A meta-analysis of the EBCTCG showed efficacy of adjuvant tamoxifen for both pre- and postmenopausal patients (EBCTCG et al. 2011). A further treatment option for premenopausal patients is the combination of tamoxifen with gonadotropin-releasing hormone (GnRH) analogs (Baum et al. 2006; Cuzick et al. 2007). Another approach in premenopausal women is ovarian function suppression combined with AIs, which was investigated in two recently published studies, the suppression of ovarian function trial (SOFT) and tamoxifen and exemestan trial (TEXT) trial (Pagani et al. 2014). Adjuvant treatment with exemestane plus ovarian suppression significantly reduced recurrence in premenopausal patients with HR-positive early breast cancer, as compared with tamoxifen plus ovarian suppression (Pagani et al. 2014).

Large-scale randomized controlled trials (RCTs) investigated the use of AIs as either a substitute or add-on to tamoxifen and showed a superiority of the AIs over tamoxifen in postmenopausal patients (Baum et al. 2002; Thürlimann et al. 2009; Coombes et al. 2004; Goss et al. 2003; Dowsett et al. 2010). Different strategies including AIs are possible, e.g., upfront monotherapy or switch to an AI after 2–3 years of tamoxifen or switch to tamoxifen after 2–3 years of AIs, and extended adjuvant treatment with an AI after 5 years of tamoxifen (Lux et al. 2013; Kolberg et al. 2012). Currently, the prolongation of ET beyond 5 years is discussed. The adjuvant tamoxifen: longer against shorter trial (ATLAS) showed that for ER-positive breast cancer patients continuing tamoxifen for up to 10 years rather than stopping at 5 years yielded a further reduction in recurrence and mortality, particularly after year 10 (Davies et al. 2013). A meta-analysis involving 29,138 patients and eight RCTs concluded that in ER-positive breast cancer patients extended ET beyond 5 years of tamoxifen significantly improved OS [OR 0.89; 95 % confidence interval (CI) 0.80–0.99; *P* = 0.03], breast cancer-specific survival (OR 0.78; 95 % CI 0.69–0.9; *P* = 0.0003), and relapse-free survival (OR 0.72; 95 % CI 0.56–0.92; *P* = 0.01) compared with 5 years of ET alone (Petrelli et al. 2013). However, further follow-up of the included trials is needed to confirm these results.

Despite these encouraging findings from numerous RCTs and meta-analyses, data on the performance of ET under routine conditions are scarce. Thus, the aim of this study was to evaluate the routine quality of diagnosis and adjuvant endocrine treatment of steroid hormone receptor (HR)-positive breast cancer patients in a large cohort of more than 7000 patients by analyzing data from a population-based regional cancer registry.

## Methods

### Database

In the present study, data from the Tumor Center Regensburg (Bavaria, Germany) were analyzed. This high-quality population-based regional cancer registry was founded in 1991 and covers a population of more than 2.2 million people of Upper Palatinate and Lower Bavaria. Currently, follow-up data of 240,655 patients of all major cancer sites are available. Following a stringent protocol, this cancer registry obtains a cross-sectorial documentation of all breast cancer patients in the area (*n* = 10,152 patients diagnosed between 2000 and 2012). Information about diagnosis, course of disease, therapies, and long-term follow-up are documented. Patient data originate from the University Hospital Regensburg, 53 regional hospitals, and more than 1000 practicing doctors in the region. Based on medical reports, pathology, and follow-up records, these population-based data are routinely being documented and fed into the cancer registry.

### Cancer registration in Bavaria

In Bavaria, the law on the Bavarian Epidemiologic Cancer Registry (*Gesetz über das bevölkerungsbezogene Krebsregister Bayern*-*BayKRG*, as amended from time to time) allows the continuous and uniform data acquisition and processing of cancer incidences by means of an epidemiologic cancer registry. The purpose of this law is to regulate cancer control and to improve data quality of cancer epidemiology. The Bavarian Epidemiologic Cancer Registry has to provide anonymous data for scientific research.

Informed consent has to be given in accordance with the Declaration of Helsinki and is an indispensable precondition for data storage. Any physician has to adequately inform the patients about the intended or performed transmission of data to the registry. Patients also receive written information about these procedures. Each patient has the right to object data storage at any time. On the basis of this law, retrospective analyses of anonymous data require no additional ethics statement.

### Inclusion and exclusion criteria

**Fig. 1:**
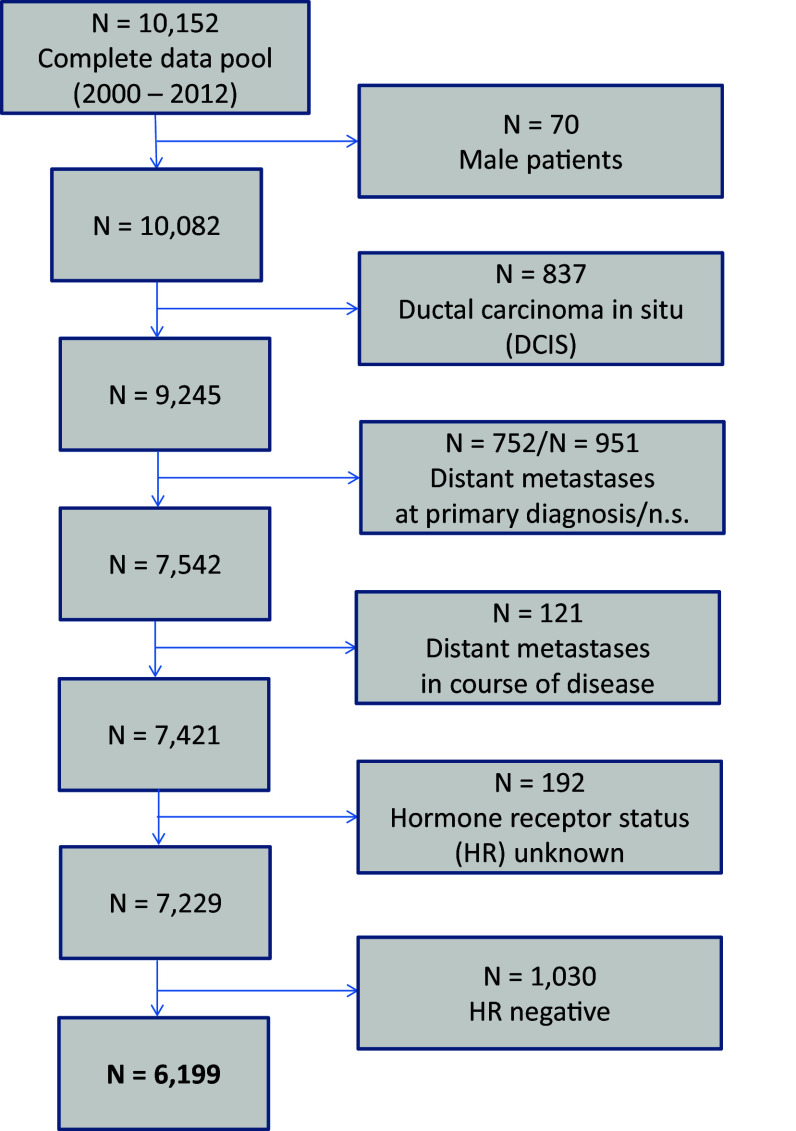
Scheme of data extraction

The present analysis included all female patients documented in the cancer registry with primary, nonmetastatic (M0) invasive breast cancer diagnosed between January 2000 and December 2012 (13 years). Follow-up data up to July 2013 were analyzed. Exclusion criteria were male patients, ductal carcinoma in situ (DCIS), and distant metastases (Fig. 1).

### Analysis of HR

Immunohistochemical determination of ER and PR was performed and quantified as the percentage of positivity of malignant cells and average intensity of coloration consistent with defined standards (Prechtel and Prechtel 2001). Additionally, the immunoreactive score (IRS) according to Remmele and Stegner (1987) was calculated. In the last update of the German interdisciplinary S3 Guideline for Diagnosis, Treatment and Follow-up Care of Breast Cancer (updated version 07/2012, registry number 032-045OL of Association of the Scientific Medical Societies, AWMF) (Kreienberg et al. 2013), cutoff definitions for the interpretation of results were modified and follow the current ASCO/CAP recommendations (Hammond et al. 2010). Since that date, at least 1 % of positively marking malignant cells is sufficient for determination of HR-status as positive. Previously, at least 10 % positivity was necessary. In the data set, HR-positivity is defined as ER+PR+, ER+PR− or ER−PR+. HR-negativity is defined as ER−PR−.

### Quality assurance methods

Overall, six institutes of pathology were involved in these analyses. Consistency and quality control are ensured through various quality assurance methods including the certification/accreditation of the pathologies according to DIN EN ISO 9001, the participation in the German interlaboratory trials, and regular regional breast cancer-specific quality assurance conferences (Inwald et al. 2013).

### Statistical analyses

Continuous data were expressed as means ± standard deviations (SD) and categorical data as frequency counts and percentages. Baseline characteristics of patients were compared between HR-status by Student’s *t* test for continuous variables and by Pearson’s Chi-square tests for categorical variables. OS was calculated from the date of cancer diagnosis to the date of death from any cause. Patients who were not dead or patients without follow-up were classified as censored. The impact of established prognostic factors (age, tumor size, nodal status, grading, HER2/neu, and menopausal status) and the extent of primary therapy on OS were assessed by means of a multivariable Cox regression analysis. Hazard ratios (*HR*) and corresponding 95 % CI were calculated and considered statistically significant if CI excluded 1.0. All reported *P* values were two-sided, and a *P* value of 0.05 was considered the threshold of statistical significance. Calculations were made with the software packages SPSS 22 (Chicago, EUA) and R (version 3.0.3).

## Results

### Analysis of patients’ characteristics

According to the ICD-10 classification, 7421 female patients with invasive, nonmetastatic breast cancer (C50) were extracted from the total data pool of breast tumor patients (Fig. 1). The HR-status was available in 97.4 % (7229 patients) (Table 1). In 2.6 % (192 patients), the HR-status was absent due to missing information in the medical reports or no analysis. Since 2009, almost a complete rate of 99.6 % of analyzed HR-status was achieved. Only patients with noted HR-status were included for further statistical evaluation. Hence, a total of 7229 breast cancer patients were considered for subsequent analyses (Table 2). In total, 1684 patients (23.3 %) were premenopausal and 5545 patients (76.7 %) were postmenopausal. Mean age was 61 years (median 61 years, range 21–97 years); 85.8 % of patients (*n* = 6199) were HR-positive, whereas 14.2 % of patients (*n* = 1030) were HR-negative. Postmenopausal women were more likely to be HR-positive (*n* = 4830, 87.1 %) than premenopausal patients (*n* = 1369, 81.3 %). All common histopathological parameters showed (highly) statistically significant differences between HR-positive and HR-negative patients. High-grade (G3) tumors were rather associated with negative HR-status. In HR-negative patients, 73.0 % of tumors were high grade compared to 20.2 % of HR-positive tumors (*P* < 0.001). HR-positive patients were more likely to be HER2-negative (75.5 %) than HR-negative patients (60.6 %) (*P* < 0.001) (Table 2). Detailed description of HR-status is shown in Table 3. The majority of patients were both ER- and PR-positive (73.9 %) in both premenopausal and postmenopausal patients.

**Table 1:** Time-dependent rates of hormone receptor (HR) analyses

Year of diagnosis	Number of patients (*n*)	HR-status unknown (*n*, %)	HR-status analyzed (*n*, %)	HR-positive (*n*, %)	HR-negative (*n*, %)
2000	448	28 (6.3 %)	420 (93.7 %)	345 (82.1 %)	75 (17.9 %)
2001	472	16 (3.4 %)	456 (96.6 %)	401 (87.9 %)	55 (12.1 %)
2002	477	19 (4.0 %)	458 (96.0 %)	379 (82.8 %)	79 (17.2 %)
2003	536	10 (1.9 %)	526 (98.1 %)	466 (88.6 %)	60 (11.4 %)
2004	595	20 (3.4 %)	575 (96.6 %)	489 (85.0 %)	86 (15.0 %)
2005	585	8 (1.4 %)	577 (98.6 %)	471 (81.6 %)	106 (18.4 %)
2006	570	1 (0.2 %)	569 (99.8 %)	487 (85.6 %)	82 (14.4 %)
2007	603	34 (5.6 %)	569 (94.4 %)	492 (86.5 %)	77 (13.5 %)
2008	608	45 (7.4 %)	563 (92.6 %)	464 (82.4 %)	99 (17.6 %)
2009	721	3 (0.4 %)	718 (99.6 %)	624 (86.9 %)	94 (13.1 %)
2010	628	4 (0.6 %)	624 (99.4 %)	542 (86.9 %)	82 (13.1 %)
2011	579	2 (0.3 %)	577 (99.7 %)	513 (88.9 %)	64 (11.1 %)
2012	599	2 (0.3 %)	597 (99.7 %)	526 (88.1 %)	71 (11.9 %)
Total	7421	192 (2.6 %)	7229 (97.4 %)	6199 (85.8 %)	1030 (14.2 %)

HR-positive is defined as ER+PR+, ER+PR−, or ER−PR+

HR-negative is defined as ER−PR−

**Table 2:** Associations between HR and clinical and histopathological parameters

Parameter	HR-positive (*n* = 6199)	HR-negative (*n* = 1030)	Total (*n* = 7229)	*P* value^a^
Age (year), mean ± SD	62 ± 13	57 ± 14	61 ± 13	<0.001
Menopausal state, *n* (%)				
Premenopausal	1369 (22.1 %)	315 (30.6 %)	1684 (23.3 %)	<0.001
Postmenopausal	4830 (77.9 %)	715 (69.4 %)	5545 (76.7 %)	
Histology, *n* (%)				
Ductal	4913 (79.3 %)	896 (87.0 %)	5809 (80.4 %)	<0.001
Lobular	876 (14.1 %)	28 (2.7 %)	904 (12.5 %)	
Other	410 (6.6 %)	106 (10.3 %)	516 (7.1 %)	
Tumor size, *n* (%)				
pT0	27 (0.4 %)	31 (3.0 %)	58 (0.8 %)	<0.001
pT1	3385 (54.6 %)	435 (42.2 %)	3820 (52.8 %)	
pT2	2217 (35.8 %)	449 (43.6 %)	2666 (36.9 %)	
pT3	237 (3.8 %)	49 (4.8 %)	286 (4.0 %)	
pT4	301 (4.9 %)	56 (5.4 %)	357 (4.9 %)	
Unknown	32 (0.5 %)	10 (1.0 %)	42 (0.6 %)	
Nodal status, *n* (%)				
pN0	3832 (61.8 %)	608 (59.0 %)	4440 (61.4 %)	0.032
pN1	1534 (24.7 %)	249 (24.2 %)	1783 (24.7 %)	
pN2	425 (6.9 %)	93 (9.0 %)	518 (7.2 %)	
pN3	283 (4.6 %)	57 (5.5 %)	340 (4.7 %)	
Unknown	125 (2.0 %)	23 (2.2 %)	148 (2.0 %)	
Grading, *n* (%)				
G1	1017 (16.4 %)	14 (1.4 %)	1031 (14.3 %)	<0.001
G2	3891 (62.8 %)	256 (24.9 %)	4147 (57.4 %)	
G3	1252 (20.2 %)	752 (73.0 %)	2004 (27.7 %)	
Unknown	39 (0.6 %)	8 (0.8 %)	47 (0.7 %)	
HER2, *n* (%)				
Negative	4678 (75.5 %)	624 (60.6 %)	5302 (73.3 %)	<0.001
Positive	952 (15.4 %)	306 (29.7 %)	1258 (17.4 %)	
Unknown	569 (9.2 %)	100 (9.7 %)	669 (9.3 %)	
Lymphatic invasion, *n* (%)				
Positive	1676 (27.0 %)	350 (34.0 %)	2026 (28.0 %)	<0.001
Negative	3024 (48.8 %)	430 (41.7 %)	3454 (47.8 %)	
Unknown	1499 (24.2 %)	250 (24.3 %)	1749 (24.2 %)	
Vascular invasion, *n* (%)				
Positive	320 (5.2 %)	92 (8.9 %)	412 (5.7 %)	<0.001
Negative	4168 (67.2 %)	634 (61.6 %)	4802 (66.4 %)	
Unknown	1711 (27.6 %)	304 (29.5 %)	2015 (27.9 %)	

HR-positive is defined as ER+PR+, ER+PR−, or ER−PR+

HR-negative is defined as ER−PR−

^a^ *P* value of *t* test or Pearson’s Chi-square test, respectively

**Table 3:** ER- and/or PR-expression

	Premenopausal (*n* = 1684, 23 %)	Postmenopausal (*n* = 5545, 77 %)	Total (*n* = 7229, 100 %)
ER+PR+	1206 (71.6 %)	4137 (74.6 %)	5343 (73.9 %)
ER+PR−	125 (7.4 %)	600 (10.8 %)	725 (10.0 %)
ER−PR+	38 (2.3 %)	93 (1.7 %)	131 (1.8 %)
ER−PR−	315 (18.7 %)	715 (12.9 %)	1030 (14.2 %)

### Distribution of HR-status across different pathologies

**Fig. 2:**
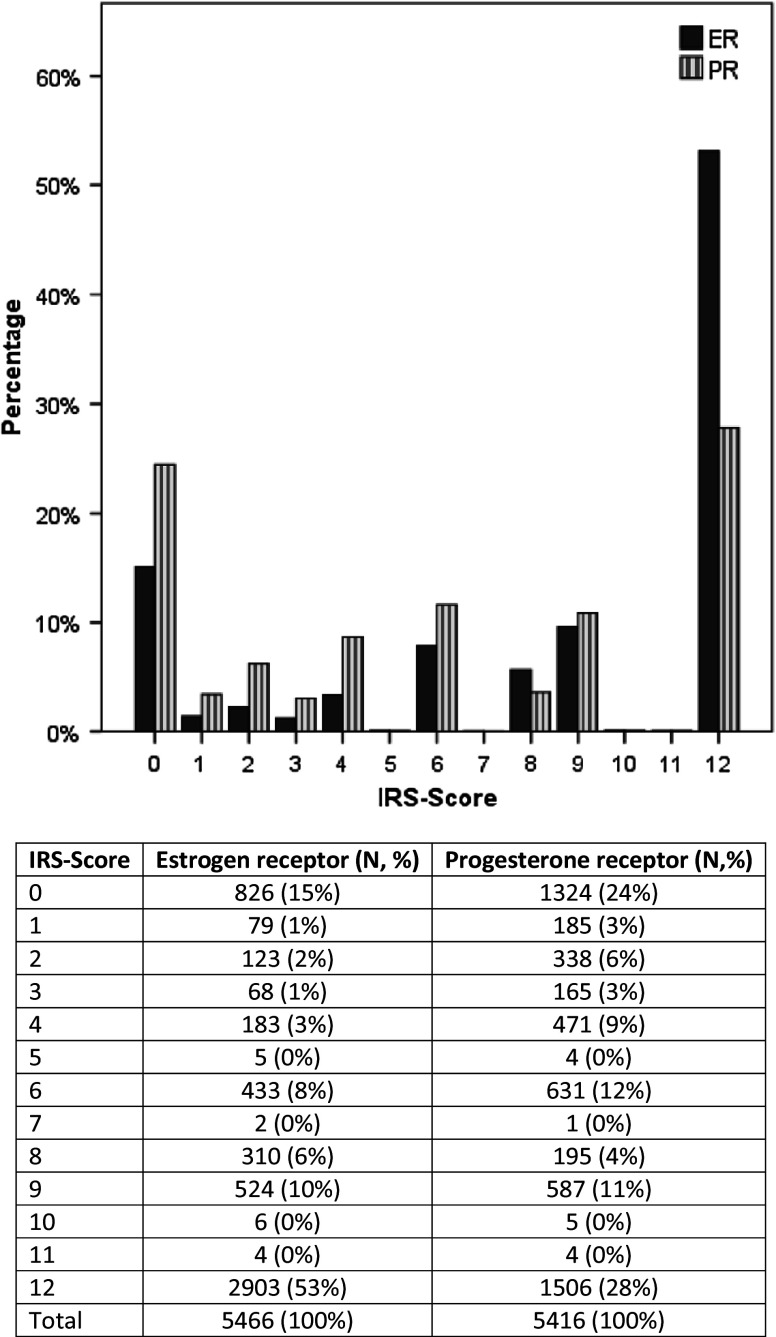
Distribution of IRS scores of ER and PR

To evaluate the inter-laboratory consistency, we investigated the distribution of patients in different institutes of pathology as well as the distribution of HR-status. A total of six institutions were involved in HR diagnostics. These analyzed samples from 133 to 1787 patients. The distribution of HR-status across the different pathologies was homogenous which reflects the established quality assurance methods in the Tumor Centre Regensburg. Regarding ER, 15 % of samples from patients (*n* = 826) had IRS 0, and 53 % (*n* = 2903) had IRS 12. With respect to PR, 24 % of patients (*n* = 1324) had IRS 0, and 28 % (*n* = 1506) had IRS 12. IRSs lying in between were underrepresented (Fig. 2).

### Systemic therapies in HR-positive patients

Overall, 5285 (85.3 %) of 6199 HR-positive patients received ET either alone or in combination with CHT and/or trastuzumab (Table 4). Thereby, the proportion of premenopausal and postmenopausal patients receiving ET was identical (85.8 % premenopausal versus 85.1 % postmenopausal).

**Table 4:** Different systemic therapies in HR-positive patients

	Premenopausal (*n* = 1369, 22.1 %)	Postmenopausal (*n* = 4830, 77.9 %)	Total (*n* = 6199, 100 %)
CHT + ET + Trastuzumab	115 (8.4 %)	170 (3.5 %)	285 (4.6 %)
CHT + Trastuzumab	14 (1.0 %)	18 (0.4 %)	32 (0.5 %)
ET + Trastuzumab	1 (0.1 %)	15 (0.3 %)	16 (0.3 %)
CHT + ET	716 (52.3 %)	1255 (26.0 %)	1971 (31.8 %)
ET	343 (25.1 %)	2670 (55.3 %)	3013 (48.6 %)
CHT	96 (7.0 %)	172 (3.6 %)	268 (4.3 %)
No adjuvant therapy	84 (6.1 %)	530 (11.0 %)	614 (9.9 %)

The majority of premenopausal patients received CHT plus ET (716 patients, 52.3 %) and ET alone (343 patients, 25.1 %), respectively. In postmenopausal patients, the most frequent systemic therapy was ET alone (2670 patients, 55.3 %) followed by CHT plus ET (1255 patients, 26.0 %). The relatively large number of 614 HR-positive patients (9.9 %) received no adjuvant therapy at all (Table 4). Further analyses regarding this revealed the following: In *n* = 368/614 (59.9 %) of these patients, an ET was planned but not started yet, *n* = 36 (5.9 %) of the patients declined an ET, and in 34.2 % of the cases, reasons for the nonuse could not be identified.

### Analysis of type of ET in HR-positive patients

Moreover, the type of ET in HR-positive patients was analyzed (Table 5). In 85.2 % of patients (*n* = 4504), the type of ET was documented. In pre- and in postmenopausal patients, the most frequently applied ET was tamoxifen (66.8 %, *n* = 666 premenopausal versus 50.5 %, *n* = 1770 postmenopausal patients). Furthermore, 20.0 % (*n* = 199) of premenopausal patients obtained tamoxifen plus GnRH and 8.7 % (*n* = 87) AIs alone. In sum, 46.3 % (*n* = 1625) of the postmenopausal patients received AIs alone. Overall, 2.5 % (*n* = 112) of patients were treated with tamoxifen followed by AI.

**Table 5:** Type of ET in HR-positive patients

	Premenopausal (*n* = 1175, 22.2 %)	Postmenopausal (*n* = 4110, 77.8 %)	Total (*n* = 5285, 100 %)
Unknown	178 (15.1 %)	603 (14.7 %)	781 (14.8 %)
Known	997 (84.9 %)	3507 (85.3 %)	4504 (85.2 %)
Tamoxifen	666 (66.8 %)	1770 (50.5 %)	2436 (54.1 %)
Tamoxifen + GnRH	199 (20.0 %)	14 (0.4 %)	213 (4.7 %)
Tamoxifen + AI	14 (1.4 %)	98 (2.8 %)	112 (2.5 %)
AI	87 (8.7 %)	1625 (46.3 %)	1712 (38.0 %)
GnRH	31 (3.1 %)	–	31 (0.7 %)

### Survival analyses in HR-positive patients

To evaluate the effects of various systemic therapies, we compared the different treatment groups (Table 6). Premenopausal patients generally showed better survival rates than postmenopausal patients. Best OS was found in HER2-/HR-positive patients receiving CHT plus ET plus trastuzumab in premenopausal as well as in postmenopausal patients (7-year OS rate of 97.2 % in premenopausal patients versus 86.9 % in postmenopausal patients). The effect of the (non-) use of trastuzumab on survival in HER2-positive breast cancer patients and its correlation with HR-status and ET has been previously shown in a study of our group in the same patient cohort (Inwald et al. 2014). In HER2-positive patients (*n* = 1258), there was a significant difference in OS between HR-positive and HR-negative patients (7-year OS rate of 83.7 % in HR-positive patients versus 76.0 % in HR-negative patients, *P* = 0.006).

**Table 6:** Overall survival rates categorized by menopausal status and adjuvant therapy

	3-Year OS (%)	5-Year OS (%)	6-Year OS (%)	7-Year OS (%)
CHT + ET + Trastuzumab				
Premenopausal (*n* = 115, 8.5 %)	98.9	97.2	97.2	97.2
Postmenopausal (*n* = 170, 3.5 %)	96.6	93.1	93.1	86.9
CHT + ET				
Premenopausal (*n* = 716, 52.9 %)	97.1	95.6	94.5	92.7
Postmenopausal (*n* = 1255, 26.2 %)	95.9	89.9	87.0	84.5
ET				
Premenopausal (*n* = 343, 25.3 %)	99.0	96.9	96.5	95.3
Postmenopausal (*n* = 2670, 55.7 %)	93.9	87.8	84.8	81.7
CHT				
Premenopausal (*n* = 96, 7.1 %)	92.7	83.7	77.6	77.6
Postmenopausal (*n* = 172, 3.6 %)	80.5	75.9	74.9	72.4
No adjuvant therapy				
Premenopausal (*n* = 84, 6.2 %)	94.1	90.8	73.1	73.1
Postmenopausal (*n* = 530, 11.0 %)	79.2	68.8	63.5	58.9

Premenopausal patients: *n* = 1354

Postmenopausal patients: *n* = 4797

Total: *n* = 6151

### Survival effects as a function of menopausal state

**Fig. 3:**
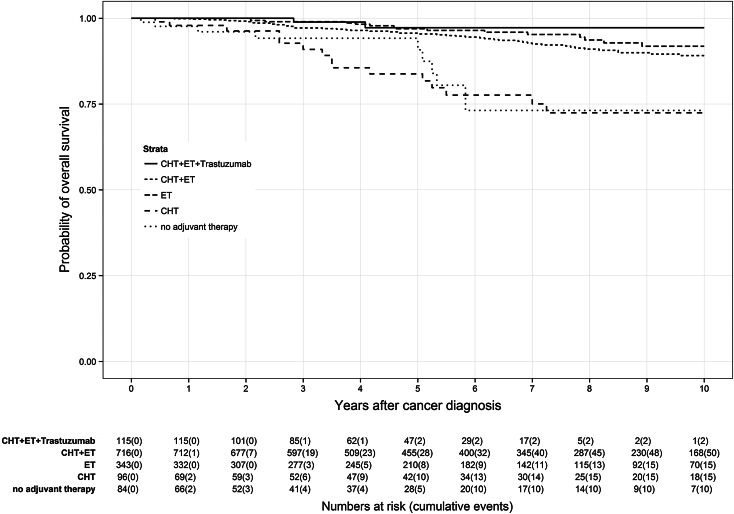
Kaplan–Meier plot of overall survival in years of premenopausal patients based on adjuvant therapy

**Fig. 4:**
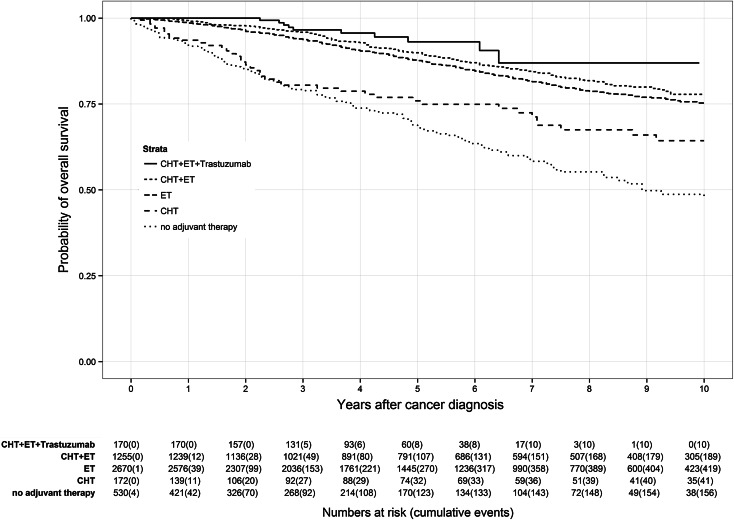
Kaplan–Meier plot of overall survival in years of postmenopausal patients based on adjuvant therapy

Application of CHT prior to ET led to different survival effects in both age groups (Figs. 3, 4). Premenopausal patients (Fig. 3) had a reduced benefit from additional CHT than postmenopausal patients (Fig. 4). Premenopausal patients receiving only ET had a 7-year OS rate of 95.3 % compared to 92.7 % of patients receiving CHT plus ET. In contrast, postmenopausal patients treated with CHT plus ET had a 7-year OS rate of 84.0 % in comparison with those patients receiving only ET with a 7-year OS rate of 81.7 %. Depriving HR-positive patients ET and only administering CHT caused lower OS rates in both age groups than in the particular control group. Lowest OS of HR-positive patients was found in patients receiving no adjuvant therapy at all with a 7-year OS rate of 73.1 % in premenopausal patients and 58.9 % in postmenopausal patients.

A Cox regression model (Table 7) provided further evidence that HR-positive patients (*n* = 5410, 705 events) without ET showed lower OS than the control groups. Patients without ET but only CHT (HR 4.54, 95 % CI 2.29–9.00, *P* < 0.001) and patients receiving no adjuvant therapy at all (HR 4.69, 95 % CI 2.43–9.06, *P* < 0.001) had the lowest OS.

**Table 7:** Multivariable Cox proportional hazard model on overall survival

Characteristic	Overall survival (*n* = 5410, events = 705)		
	HR	95 % CI	*P* value
Primary therapy			
CHT + ET + Trastuzumab	Reference		
CHT + ET	1.68	0.89, 3.15	0.109
ET	2.14	1.13, 4.06	0.019
CHT	4.54	2.29, 9.00	<0.001
No adjuvant therapy	4.69	2.43, 9.06	<0.001
Age (years)	1.05	1.04, 1.06	<0.001
Tumor size			
pT1	Reference		
pT2	1.53	1.27, 1.84	<0.001
pT3	2.06	1.49, 2.85	<0.001
pT4	2.51	1.92, 3.27	<0.001
Nodal status			
N0	Reference		
N1	1.55	1.29, 1.87	<0.001
N2	1.90	1.46, 2.48	<0.001
N3	2.69	2.04, 3.55	<0.001
Grading			
G1	Reference		
G2	1.29	0.99, 1.68	0.063
G3	2.00	1.49, 2.68	<0.001
HER2/neu			
Negative	Reference		
Positive	1.00	0.81, 1.23	0.986
Menopausal status			
Premenopausal	Reference		
Postmenopausal	0.67	0.48, 0.92	0.012

## Discussion

National and international guidelines strongly recommend the determination of HR-status in all patients with invasive breast cancer (Kreienberg et al. 2013; Untch et al. 2013). In HR-positive early breast cancer, adjuvant ET is considered as standard care. Using data from a high-quality population-based regional cancer registry, we were able to analyze the quality of routine care.

HR determination steadily increased from 93.7 % in 2000 to 99.7 % in 2012 and from 2009 on reached a constant peak of 99.6 %. This demonstrates the high quality of HR assessment in the investigated area. Concerning HR-status, a slight increase in HR-positive breast cancer was observed from 82.1 % in 2000 to 88.1 % in 2012. In HR-positive breast cancer, the most common type was both ER- and PR-positive tumors with 71.6 % in premenopausal patients and 74.6 % in postmenopausal patients. Inter-laboratory consistency between the pathologies was given as the HR-status displayed to be homogenous. This fact can be expressed through the established quality assurance methods in the Tumor Centre Regensburg. The majority of breast cancers were scored as IRS 0 and IRS 12, whereas the IRSs lying in between were underrepresented in both ER and PR analyses. This distribution of IRS might be a function of tumor biology. In total, 73.9 % of patients were ER + PR + which represent Luminal A patients regarding the biology of tumors. These patients usually show high expression of ER and PR. By contrast, triple-negative tumors, i.e., basal-like tumors, are characterized by HR-negativity, defined as ER−PR−. In total, 14.2 % of our patients belong to this group. Analysis of systemic therapies showed that 85.3 % of all HR-positive patients received ET either alone or in combination with CHT and/or trastuzumab which is in accordance with prior analyses of clinical cohort studies (Van Ewijk et al. 2013). However, nearly 10 % of HR-positive patients did not receive adjuvant therapy. This finding is comparable to data from a longitudinal study of breast cancer patients reported to the Metropolitan Detroit and Los Angeles SEER cancer registries: Of the 743 patients eligible for ET, 10.8 % never initiated therapy and 15.1 % started therapy but discontinued prematurely (Friese et al. 2013). Up to 5 % of HR-positive patients did not receive ET but only CHT. This might be due to lacking compliance of these patients. Generally, adjuvant therapy begins with CHT followed by ET. Presumably, these patients declined ET as they had to suffer from many side effects from CHT. They might worry about further side effects from ET and the long duration of therapy.

The most frequently applied form of systemic treatment was a combination of CHT and ET in premenopausal and ET alone in postmenopausal patients. However, only postmenopausal patients had a distinct benefit from the addition of CHT prior to ET. The Oxford Overview (Pritchard et al. 2012) similarly showed that there was a trend suggesting greater CHT effect in the age 55- to 69-year group than in younger women. A Danish study from the population-based database of the Danish Breast Cancer Cooperative Group evaluated 6529 postmenopausal patients with ER-positive high-risk breast cancer and also concluded that only one quarter of postmenopausal patients are free of excess mortality when omitting adjuvant CHT (Ejlertsen et al. 2014). A 10-year update of the International Breast Cancer Study Group (IBCSG) Trial 11-93 demonstrated no evidence that adjuvant CHT provides additional disease control for premenopausal patients with lower-risk node-positive endocrine-responsive breast cancer who receive adequate adjuvant ET (Thürlimann et al. 2009). The reasons for these therapy effects in different age groups are probably multifactorial. A possible explanation might be a bias of selected therapies. It can be presumed that only patients without comorbidities, which are found more often in premenopausal patients, get an additional CHT. This observation has already been made in a previous study of our group (Inwald et al. 2014). Another explanation might be incompliant long-term use of ET especially in postmenopausal patients (Demissie et al. 2001). It has been shown that the adherence to adjuvant therapy in clinical practice is relatively poor, with up to 50 % of women not completing ET (Chlebowski et al. 2014). A study from an online breast cancer research registry showed that nonadherence among users was significantly associated with a lower financial status, a poorer relationship with the oncologist, and a prior switch in endocrine therapies (Stanton et al. 2014). However, low adherence to adjuvant ET increases the risk of death (Makubate et al. 2013). Nevertheless, there are some limitations of this population-based study: These data primarily cover subnational or regional districts and might not reflect the entire population. The regional data might not be representative of international data due to regional variances, e.g., risk factors or access to early screening. However, the strength of the data is that it reflects routine healthcare provisions. Consequently, these data can be used to analyze the structures of patient-centered care.

## Conclusions

We conclude that analysis of HR in patients with early breast cancer achieved very high quality in recent years due to implementation of guidelines and control mechanisms. In line with current guidelines, the vast majority of HR-positive patients mostly received ET and this resulted in improved OS. Furthermore, our study showed differential effects of CHT and ET combination in premenopausal versus postmenopausal patients not previously described in a population-based cohort. In light of this positive finding, it is of major importance to track the minority of patients who did not receive appropriate therapy and to identify the reasons for this fatal deviation from current guidelines.

## Abbreviations

ET: Endocrine therapy
HR: Hormone receptor
CHT: Chemotherapy
ER: Estrogen receptor
PR: Progesterone receptor
AIs: Aromatase inhibitors
OS: Overall survival
GnRH: Gonadotropin-releasing hormone
RCTs: Randomized controlled trials
IRS: Immunoreactive score
SD: Standard deviation
CI: Confidence interval
